# Developmental Changes of TGF-β1 and Smads Signaling Pathway in Intestinal Adaption of Weaned Pigs

**DOI:** 10.1371/journal.pone.0104589

**Published:** 2014-08-29

**Authors:** Kan Xiao, Ze-He Song, Le-Fei Jiao, Ya-Lu Ke, Cai-Hong Hu

**Affiliations:** Animal Science College, Zhejiang University, The Key Laboratory of Molecular Animal Nutrition, Ministry of Education, Hangzhou, China; Qingdao Agricultural University, China

## Abstract

Weaning stress caused marked changes in intestinal structure and function. Transforming growth factor-β1 (TGF-β1) and canonical Smads signaling pathway are suspected to play an important regulatory role in post-weaning adaptation of the small intestine. In the present study, the intestinal morphology and permeability, developmental expressions of tight junction proteins and TGF-β1 in the intestine of piglets during the 2 weeks after weaning were assessed. The expressions of TGF-β receptor I/II (TβRI, TβRII), smad2/3, smad4 and smad7 were determined to investigate whether canonical smads signaling pathways were involved in early weaning adaption process. The results showed that a shorter villus and deeper crypt were observed on d 3 and d 7 postweaning and intestinal morphology recovered to preweaning values on d 14 postweaning. Early weaning increased (*P*<0.05) plasma level of diamine oxidase (DAO) and decreased DAO activities (*P*<0.05) in intestinal mucosa on d 3 and d 7 post-weaning. Compared with the pre-weaning stage (d 0), tight junction proteins level of occludin and claudin-1 were reduced (*P*<0.05) on d 3, 7 and 14 post-weaning, and ZO-1 protein was reduced (*P*<0.05) on d 3 and d 7 post-weaning. An increase (*P*<0.05) of TGF-β1 in intestinal mucosa was observed on d 3 and d 7 and then level down on d 14 post-weaning. Although there was an increase (*P*<0.05) of TβR II protein expression in the intestinal mucosa on d3 and d 7, no significant increase of mRNA of TβRI, TβRII, smad2/3, smad4 and smad7 was observed during postweaning. The results indicated that TGF-β1 was associated with the restoration of intestinal morphology and barrier function following weaning stress. The increased intestinal endogenous TGF-β1 didn't activate the canonical Smads signaling pathway.

## Introduction

Weaning is the most significant event in the life of pigs as they are abruptly forced to adapt to nutritional, immunological and psychological disruptions. An abundance of researches have reported that early weaning causes marked changes in intestinal structure and function such as villus atrophy, crypt hyperplasia, decreased digestive and absorptive capacity and impaired intestinal barrier [1]–[5]. It was reported that the most severe reduction in villus was observed in pigs 2-5 d post-weaning and the morphology recovered to preweaning values on d 14 postweaning [4], [6]. Particularly, the recovery of intestinal barrier function was slower than that of intestinal mucosal morphology [4]. After injury, mucosal repair is a complex event that included epithelial cells adjacent to the injured surface migrating into the wound and then epithelial cells proliferation, maturation and differentiation [7], [8]. This is a highly regulated event involving multiple growth factors in the restoration of damaged intestine [7], [8].

Transforming growth factor-β has been suspected to be an important modulator of the intestinal development and function in postnatal pigs [9]–[11]. Mei et al. (2005) found transient changes of TGF-β1 expression and distribution in the small intestine of the pig during weaning and speculated that TGF-β1 played an important regulatory role in restoration of intestinal structure. TGF-β is a multifunctional polypeptide growth factor which has a central role in modulating gut mucosal cell growth, differentiation, migration and epithelial restitution [7], . The TGF-β signaling pathway is mediated by smads family proteins, which transduce signals from the cell surface directly to the nucleus to regulate target gene transcription [13]. The role of TGF-β1 in restoration of barrier integrity has been demonstrated *in vitro* [14]–[16]. However, little data is available about developmental changes of TGF-β1, it receptors and canonical smads signaling pathway in weaning pigs.

Therefore, the present study was aimed to gain more insight into the developmental changes of TGF-β1, it receptors (TGF-β receptor I, II), smads signaling pathway and tight junction proteins (Occludin, Claudin-1 and ZO-1) in weaned pigs. It would be of interest to determine the involvement of smads signaling pathway in the role of TGF-β1 in intestinal barrier of weaned piglets.

## Materials and Methods

All procedures were approved by the Zhejiang University Animal Care and Use Committee.

### Animals, housing and diet

All procedures were according to Hu et al. [4]. Six litters (Duroc×Landrace×Yorkshire, 9 to 11 piglets per litter) were selected. At 20 days of age (preweaning stage), one piglet from each of six different litters was killed. At weaning day (21 days of age), three piglets from each of six different litters were allocated to one of the three experimental groups killed at 3, 7 and 14 d postweaning. For each group, six piglets from six different litters were removed from the sow, mixed and housed in nursery pens. The three pens have equal numbers of males and females, with average body weight of the piglets (mean ± SE, 5.7±0.2 kg). The weaned piglets were given *ad libitum* access to feed and water. The ingredient and chemical composition of the weaned diet were as described by Hu et al. [4], which was formulated to meet requirements suggested by the NRC (1998).

### Sequential killing and sample collection

On the day of preweaning (d 0) and d 3, 7 and 14 postweaning, six piglets were slaughtered respectively as described by Hu et al. [4]. Blood samples were taken from the anterior vena cava into tubes containing sodium heparin and mixed immediately to avoid coagulation. Plasma was obtained after centrifugation at 3000×g for 15 min at 4°C and then stored at −80°C for analysis. The intestinal tract was removed immediately. Specimens of mid-jejunum were fixed in buffered formalin until morphology measurements. Adjacent mucosal scrapings were collected, rapidly frozen in liquid nitrogen, and stored at −80°C.

### Intestinal morphological analysis

The specimens of mid-jejunum were embedded in paraffin, sectioned (5 µm), and stained with hematoxylin-eosin. Villus height and crypt depth were determined using an image processing and analysis system (Version 1, Leica Imaging Systems Ltd., Cambridge, UK).

### DAO activity in plasma and intestinal mucosa

The levels of diamine oxidase (DAO; EC 1.4.3.6) in plasma and intestinal mucosa were measured using an enzymatic spectrophotometric assay as described by Hu et al. [35]. Cadaverine dihydrochloride, o-dianisidine dihydrochloride, peroxidase from horseradish and DAO standard were purchased from Sigma Chemical Company (St. Louis, MO, USA). DAO activities in plasma and intestinal mucosa were expressed as U/mL and U/mg protein, respectively.

### Western blot analysis

The total levels of tight junction proteins (Occludin, Claudin-1, ZO-1, TGF-β1, TGF-β receptor I, II) in jejunal mucosa were analyzed by Western blot as previously described [11], [36], [39]. Briefly, after electrophoresis the proteins were transferred to polyvinylidene difluoride membrane (Millipore, Bed-ford, MA, USA). The following primary antibodies were used (Santa Cruz biotechnology, Santa Cruz, CA, USA): Occludin, rabbit IgG; Claudin-1, rabbit IgG; ZO-1, rabbit IgG; TGF-β1, rabbit IgG; TGF-β receptor I, rabbit IgG; TGF-β receptor II, rabbit IgG. The secondary antibody was horseradish peroxidase (HRP)-conjugated anti-rabbit IgG (Santa Cruz biotechnology, Santa Cruz, CA, USA). An enhanced chemiluminescence detection kit (Amersham, Arlington Heights, IL, USA) was used to detect the positive bands. The values in samples from the preweaning (d 0 postweaning) pigs were used as the reference sample. The protein expression of all samples was expressed as fold changes, calculated relative to the values from the preweaning (d 0 postweaning) pigs.

### mRNA expression of smads by real-time PCR

The mRNA expressions of TGF-β receptor (I, II) and smads (smad2, smad3, smad4, smad7) from jejunal mucosa were determined by quantitative real-time Polymerase Chain Reaction (qRT-PCR) as described by Liu et al. [37], [40]. The Genbank accession numbers, sequences of forward and reverse primers, and fragment sizes are presented in Table 1. Briefly, total RNA was isolated using the TRIzol Reagent (Invitrogen, Carlsbad, CA, USA) and treated with RNase-free DNase I prior to cDNA synthesis following the manufacturer's guidelines. The qRT-PCR was performed on a StepOne Plus real-time PCR system (Applied Biosystems, Foster, CA, USA) using a SYBR Green Master mix (Promega, Madison, WI, USA) according to the kit's instructions. The housekeeping gene, GAPDH, exhibited no variation across treatment groups. The preweaning (d 0 postweaning) pigs were used as the reference sample. The 2^−ΔΔCt^ method [38] was used to analyze the relative expression (fold changes), calculated relative to the values from the preweaning (d 0 postweaning) pigs.

**Table 1:** Genbank accession numbers, sequences of forward and reverse primers, and fragment sizes used for real-time PCR.

Primer name	Primer sequence	Size(bp)	Accession numbers
TβR I	F:5′CTGTGTCTGTCCACCATTCATTTG3'	496	AF461808
	R:5′CAACTTTGCTATGTCTGTCTCCCC3′		
TβR II	F:5′CATCTCCTGCTAATGTTGTTGCC3'	324	X70142
	R:5′CGGTTCTAAATCCTGGGACACG3′		
Smad2	F:5′GAAGAGAAGTGGTGTGAGAAAGCAG3'	428	BP437096
	R:5′AATACTGGAGGCAAAACTGGTGTC3′		
Smad3	F:5′TGGAGGAGGTGGAGAAATCAGAAC3'	541	AB052738
	R:5′CACACTCGCTTGCTCACTGTAATC3′		
Smad4	F:5′CCTGAGTATTGGTGTTCCATTGC3'	598	NM 214072
	R:5′TGATGCTCTGCCTTGGGTAATC3′		
Smad7	F:5′TACTGGGAGGAGAAGACGAGAGTG3'	241	AW359979
	R:5′TGGCTGACTTGATGAAGATGGG3′		
GAPDH	F:5′ATGGTGAAGGTCGGAGTGAAC3'	235	NM001206359
	R:5′CTCGCTCCTGGAAGATGGT3′		

### Statistical analysis

Statistical analyses were performed with the SAS software package (version 8.1; SAS Institue, Cary, NC, USA) (SAS, 2000). Data were subjected to one-way analysis of variance followed by Duncan's multiple range tests. Differences were considered statistically significant at *P*<0.05.

## Results

### Intestinal morphology and barrier function

Table 2 shows jejunal morphology and barrier function of piglets at different time points (0, 3, 7 and 14 d postweaning). Compared with the preweaning stage (d 0 postweaning), villus height and the ratio of villus height and crypt depth on d 3 and d 7 postweaning were decreased (*P*<0.05). However, these parameters had no significant difference (*P*>0.05) between on d 0 and d 14 postweaning. Intestinal barrier function of weaned piglets is reflected by DAO activity in plasma and intestinal mucosa in Table 3. As compared with the control, the level of DAO increased (*P*<0.05) in plasma on d 3 and d 7. However, the level of DAO in intestinal mucosa decreased (*P*<0.05) on d 3 and d 7 significantly and it recovered to the preweaning values on d 14.

**Table 2:** Intestinal mucosal morphology of piglets after weaning.

	Day post-weaning				S.E.M^1^
	0	3	7	14	
Villus height, µm	765^a^	452^b^	468^b^	731^a^	24.6
Crypt depth, µm	274^b^	316^a^	320^a^	285^b^	11.2
Villus height:crypt depth	2.8^a^	1.4^b^	1.5^b^	2.6^a^	0.11

ab Means within a row with different letters differ significantly (*P*<0.05). Data are means of six pigs.

1 Standard error of the mean, n = 6.

**Table 3:** Diamine oxidase (DAO) in plasa and mucosa of weaned pigs.

	Day post-weaning				S.E.M^1^
	0	3	7	14	
Jejunal mucosa DAO, U/mg protein	0.21^a^	0.15^b^	0.16^b^	0.18^ab^	0.01
Plasma DAO, U/mL	1.08^c^	1.65^a^	1.51^ab^	1.24^bc^	0.08

abc Means within a row with different letters differ significantly (*P*<0.05). Data are means of six pigs.

1 Standard error of the mean, n = 6.

### Tight junction proteins expression

**Figure 1:**
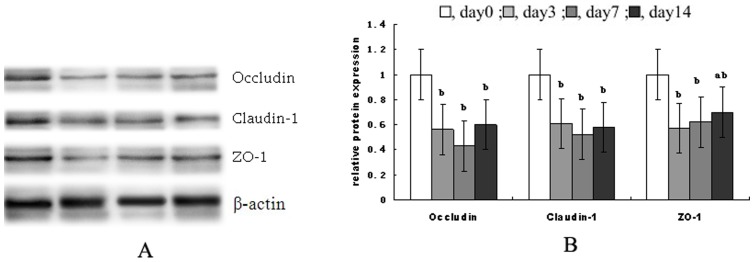
Tight junction protein levels of occludin, claudin-1 and zonula occludens-1 (ZO-1). (A) shows representative blots of occludin, claudin, zonula occludens-1 (ZO-1), and β-actin on 0, 3, 7 and 14 post-weaning, respectively. (B) shows relative tight junction proteins expression. Data are means ± SD. ^a,b^Means with different letters differ significantly (*P*<0.05). The control sample on day 0 post-weaning was used as the reference sample. The protein expression of all samples was expressed as fold changes, calculated relative to the control group on day 0 post-weaning.

Fig. 1 shows the tight junction protein levels of occludin, claudin-1 and zonula occludens-1 (ZO-1) in jejunal mucosa during the 2 weeks after weaning. Compared with the preweaning stage (d 0 post-weaning), protein levels of occludin and claudin-1 on d 3, d 7 and d 14 post-weaning and ZO-1 protein on d 3 and d 7 post-weaning were decreased significantly (*P*<0.05).

### TGF-β1 expression

**Figure 2:**
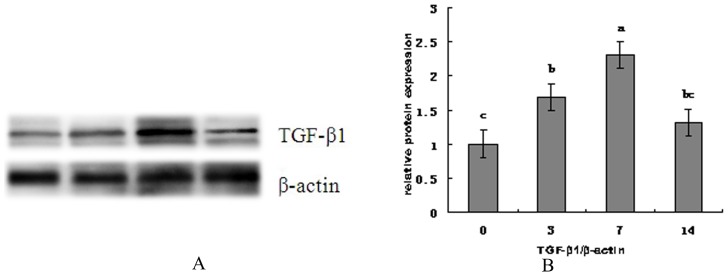
The change of TGF-β1 level in jejunal mucosa of weaned piglets during the 2 weeks. (A) shows representative blots of TGF-β1 expression and β-actin on 0, 3, 7 and 14 post-weaning, respectively. (B) shows relative TGF-β1 protein expression in jejunal mucosa of piglets after weaning. ^a,b,c^ Mean values with unlike letters were significantly different (*P*<0.05). Values are means and standard deviations represented by vertical bars. The control sample on day 0 post-weaning was used as the reference sample. The protein expression of all samples was expressed as fold changes, calculated relative to the control group on day 0 post-weaning.

Fig. 2 shows the change of TGF-β1 level in jejunal mucosa of weaned piglets during the 2 weeks. Compared with the preweaning stage (day 0), a significant increase (*P*<0.05) in protein abundance of TGF-β1 was observed on d 3 and d 7 and then level down to the normal level on d 14.

### TGF-β receptors expression

**Figure 3:**
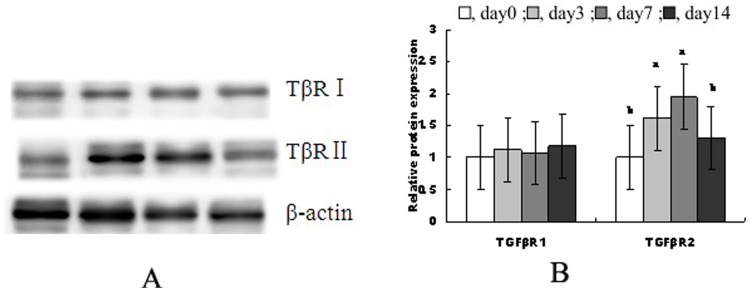
The protein levels of TβRI and TβRII in jejunal mucosa during the 2 weeks after weaning. (A) shows representative blots of TβR I, TβR II proteins expression and β-actin on 0, 3, 7 and 14 post-weaning, respectively. (B) shows relative TβR I and TβR II proteins expression. ^a,b,^Mean values with unlike letters were significantly different (*P*<0.05). Values are means and standard deviations represented by vertical bars. The value of protein expression was the ratio of the densitometry units of TβR I, TβR II protein and β-actin. The control sample on day 0 post-weaning was used as the reference sample. The protein expression of all samples was expressed as fold changes, calculated relative to the control group on day 0 post-weaning.

Fig. 3 shows protein levels of TβRI and TβRII in jejunal mucosa during the 2 weeks after weaning. Compared with the preweaning stage (day 0), TβRI did not vary significantly over time after weaning, but TβRII increased significantly on d3 and d7 (*P*<0.05) compared to day 0 postweaning.

The mRNA expressions of TβRI and TβRII in jejunal mucosa during the 2 weeks after weaning were showed in table 4. There were no significant changes in the amount of TβRI and TβRII mRNA over time after weaning.

**Table 4:** mRNA expressions of smads signals in weanling pigs.^1^

Day postweaning					
Item	0	3	7	14	SEM^2^
TβR I	1.00	1.25	1.36	1.31	0.20
TβR II	1.00	1.37	1.28	1.41	0.22
Smad2	1.00	1.20	1.31	1.28	0.14
Smad3	1.00	1.18	1.23	1.34	0.12
Smad4	1.00	1.41	1.28	1.37	0.15
Smad7	1.00	1.26	1.24	1.35	0.12

Means within a row with different letters differ significantly (*P*<0.05).

1 The 2^−ΔΔCt^ method was used to analyze the relative expression (fold changes), calculated relative to the values in samples from the preweaning (d 0 postweaning) pigs. Data are means of six pigs.

2 Standard error of the mean.

### mRNA expression of smads

Table 4 shows the mRNA expression of smads signal components in jejunal mucosa during the 2 weeks after weaning. Compared with the pre-weaning stage (day 0 post-weaning), there were no significant changes in amounts of mRNA for the smad2, smad3, smad4 and smad7 over time post-weaning.

## Discussion

Weaning piglets are abruptly forced to combined stressors, such as removal from sow and littermates, transportation to a new environment and abrupt changes in diet [1]. Early weaning stress in pigs has been reported to impair intestinal architecture and function leading to gut-associated disorders and diarrhea [4], [17]–[19]. In agreement with earlier reports, the present study showed that villus height and the ratio of villus height and crypt depth on d 3 and d 7 postweaning decreased significantly and returned to preweaning value on d 14 postweaning compared with the preweaning stage. In the present experiment, plasma and mucosa DAO was used to reflect the destruction of the intestinal mucosa barricade [20]. Diamine oxidase is found exclusively in small intestine. When intestinal mucosal barrier is damaged, intestinal mucosal cells underwent necrosis and sloughed off into the intestinal lumen which will lead to a decrease in intestinal mucosal DAO and an increase in circulating level of DAO [20], [21]. A significant increase in plasma DAO and a decrease in intestinal mucosa DAO were observed on d 3 and d 7 and plasma and mucosa DAO returned to the preweaning values on d 14 compared with d 0 in the present study. We also analyzed the expressions of inter-epithelial tight junctions, such as claudins, occludins and zonula occludens-1. There is a significant decrease of occludin and claudin-1 on d 3, d 7 and d 14 and ZO-1 on d 3 and d 7 after weaning compared with d 0, which indicated that early weaning induced sustained impairment in intestinal barrier characterized by decreased expression of tight junction protein. However, while it gradually recovered to the preweaning level on d 14. The temporal changes induced in the small intestine by weaning can be divided into two periods: an acute period happening immediately after weaning, followed after day 5 by a more progressive adaptative and maturational phase. In acute period, dramatic compromising alterations in villus-crypt structure and function are common in weaned pigs. After 5 day post-weaning, the intestine began to repair until about two weeks post weaning. After injury, villus contraction is the initial phase of repair and is initiated by myofibroblasts that reside immediately beneath the epithelial basement membrane. Subsequent events include crawling of healthy epithelium adjacent to the wound, referred to as restitution. Finally, increased proliferation and differentiation of multi-potential stem cells within crypts migrated along the crypt-villus axis for replacement of lost cells and maintenance of normal intestinal epithelial architecture and function [8].This is a highly regulated event involving multiple growth factors in the restoration of damaged intestine [7], [8].

TGF-β1 is believed to play an important regulatory role in post-weaning adaptation process in the intestine of the pig [6]. TGF-β1 plays an important regulatory role in mucosal immune reactions and intestinal barrier restoration. During injury or disease, TGF-β stimulates epithelial cell migration, increases extracellular matrix and integrin production, promotes intestinal epithelial restitution, and improves intestinal mucosa integrity [15], [16], [22]. An earlier study discovered that TGF-β induced epithelial barrier enhancement and identified TGF-β as an agent capable of blocking Enterohemorrhagic Escherichia coli O157:H7-induced increases in epithelial permeability [15]. It is also reported that TGF-β1 preserves intestinal epithelial barrier function and promotes intestinal epithelial restitution after exposure to agents known to cause barrier disruption, such as IFN-γ, and infection with *Cryptosporidium parvum* and enterohemorrhagic *E. coli* [14]–[16]. Mei et al. (2005) showed that the expression intensity of TGF-β1 at the intestinal villus epithelium decreased significantly 4 d after weaning. The transient decline in TGF-β1 level of the intestinal villus following weaning contributed to the post-weaning intestinal villus atrophy [6]. However, the role of TGF-β1 in restoration of mucosal barrier integrity has only been demonstrated *in vitro*. So far, the developmental expression of TGF-β1 on weaning pigs epithelial barrier is little available. In the present study, expression of TGF-β1 in the small intestine increased significantly on d 7 and then leveled down on d 14. In accordance with the present finding, an earlier study in the rat showed that the endogenous production of TGF-β1 in the small intestine of the pup increased significantly after midweaning [23]. Other authors showed that TGF-β1 was increased in the lamina propria cells in inflamed mucosa [24]. The increased level of TGF-β1 may stimulate the migration of epithelial cells from the wound margin and enhance rapid intestinal epithelial restitution or stimulate the synthesis of extracellular matrix proteins [12]. These events have important implications for intestinal epithelial cells intercellular tight junctions, for its growth on matrix proteins composing the basement membrane and ultimately for its barrier function.

The canonical TGF-β signaling pathway is mediated by smads family proteins. When TGF-β reach the membrane of target cells, they bind directly to TGFβ type II receptors (TβRII), which leads to the recruitment of TGFβ type I receptors (TβRI), TβRII then trans-phosphorylates TβRI, enabling the TβRI kinase domain to act on cytoplasmic proteins and thereby propeling downstream signaling actions. Following stimulation by TGF-β, Smad2 and Smad3 become phosphorylated. Phosphorylated Smad 2/3 can complex with Smad4 (the common-mediator Smad), and then translocate to the nucleus and regulate gene expression [13]. The key factors for regulating signaling component stability and balancing the incoming TGFβ signal are the inhibitory (I-) Smads (smad7) [25]. In the present study, TβRI protein abundance and mRNA expression, mRNA expressions of smad2/3, smad4 and smad7 in the intestinal mucosa did not vary significantly compared to preweaning values; However, TβR-βII protein abundance expression elevated on d 3 and d 7, then decreased to the preweaning level on d 14. The different degree expression for different receptors in the small intestine may allow more precise control of signal transduction during development. Smads protein, the only substrates of TβR (TGF-β receptor) kinase, are critical mediators of TGF-β signaling transducer. The changes of smads protein expression may partially indicate the activation of TGF-β/Smads signaling pathways [26], [27]. In the present study, there is no mRNA expression change of smad2/3, smad4 and smad7 in weaning piglets, associated with unchanged TβRI, which may suggested the canonical smads pathway was not activated by weaning stress. The notion that cross-talk with other pathways plays a defining role in how TGF-β superfamily signals in postweaning adaption are remained to be read and interpreted.

Several studies reported that TGF-β could activate Smad-independent pathways [28]–[30]. Martin-Martin et al. (2011) reported that cyclosporine A-induced increased TGF-β1 may not be sufficient to trigger the Smad pathway but may trigger the ERK1/2 signaling pathway. Other authors reported that TGF-β1 induced A549 alveolar epithelial cells to undergo epithelial-mesenchymal transition partially via p38 MAPK and JNK activation in lung epithelial cells [31]. There is evidence that the activation of the ERK 1/2 signaling is linked to the TGF-β1 induced modulation of tight junction permeability and wound closure [32], [33]. The ERK has been reported to regulate the integrity of tight junction and directly interacted with occludin to prevent H_2_O_2_-induced disruption of tight junctions [34]. Our previous study has discovered that early weaning activated MAPK signaling pathways in the intestine of weaning piglets [4], [41], the data from this study demonstrated that TGF-β1 canonical signaling pathway did not vary significantly over time. We speculated that increased endogenous TGF-β1 may trigger noncanonical MAPK signaling pathway to involve in post-weaning adaption.

In summary, the present study showed that intestinal morphology and barrier were damaged on d 3 and d 7 postweaning and recovered on d 14 postweaning. Tight junction proteins of occludin and claudin-1 were reduced on d 3, 7 and 14 post-weaning, and ZO-1 protein was reduced on d 3 and d 7 post-weaning. TGF-β1 experienced an increase on d 3 and d 7 and then returned to the preweaning value on d 14, while canonical TGF-β signaling pathway did not activated. In view of the aforementioned observations, we hypothesized that TGF-β1 involved in post-weaning adaption was Smad-independent pathways, possibly through the MAPK signalling pathways. The mechanism involved in this process needs to be further investigated.
